# Impact of Arginine–Phosphate Interactions on the Reentrant Condensation of Disordered Proteins

**DOI:** 10.1021/acs.biomac.0c01765

**Published:** 2021-03-18

**Authors:** Samuel Lenton, Stefan Hervø-Hansen, Anton M. Popov, Mark D. Tully, Mikael Lund, Marie Skepö

**Affiliations:** † Theoretical Chemistry, Lund University, P.O. Box 124, SE-221 00 Lund, Sweden; ‡ BM29 BIOSAXS, European Synchroton Radiation Facility, 71 avenue des Martyrs, Grenoble, Isère 38043, France; § LINXS—Lund Institute of Advanced Neutron and X-ray Science, Scheelevägen 19, SE-223 70 Lund, Sweden

## Abstract

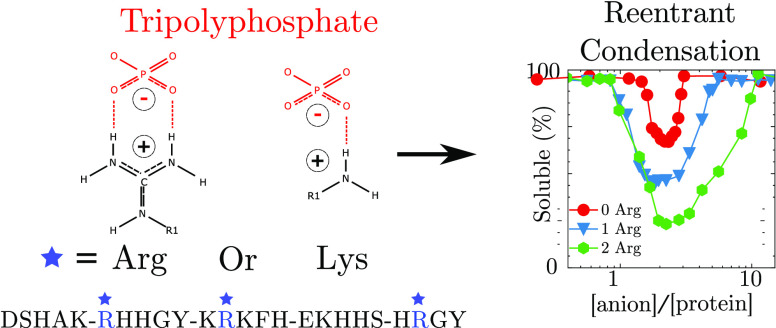

Re-entrant condensation results in the formation of a condensed protein regime between two critical ion concentrations. The process is driven by neutralization and inversion of the protein charge by oppositely charged ions. Re-entrant condensation of cationic proteins by the polyvalent anions, pyrophosphate and tripolyphosphate, has previously been observed, but not for citrate, which has similar charge and size compared to the polyphosphates. Therefore, besides electrostatic interactions, other specific interactions between the polyphosphate ions and proteins must contribute. Here, we show that additional attractive interactions between arginine and tripolyphosphate determine the re-entrant condensation and decondensation boundaries of the cationic, intrinsically disordered saliva protein, histatin 5. Furthermore, we show by small-angle X-ray scattering (SAXS) that polyvalent anions cause compaction of histatin 5, as would be expected based solely on electrostatic interactions. Hence, we conclude that arginine–phosphate-specific interactions not only regulate solution properties but also influence the conformational ensemble of histatin 5, which is shown to vary with the number of arginine residues. Together, the results presented here provide further insight into an organizational mechanism that can be used to tune protein interactions in solution of both naturally occurring and synthetic proteins.

## Introduction

The interactions between proteins in solutions are governed by a delicate balance of attractive and repulsive forces.^1,2^ A range of additives are used to tune the properties of protein solutions, including salt, and small organic molecules such as amino acids.^3,4^ The addition of salt to protein solutions can also increase protein–protein interactions, resulting in, for example, precipitation, aggregation, or oligomerization.^5−7^ The precise influence of ionic species on the interactions between proteins in solution is nontrivial and dependent on the concentration and specific properties of both the ion and the protein.^8,9^ Multivalent ions have been shown to modulate protein–protein interactions when added to solutions of oppositely charged proteins, resulting in rich phase behaviors including re-entrant condensation (RC) and liquid–liquid phase separation.^10−15^

Originally observed in solutions of anionic proteins in the presence of trivalent cations, RC is characterized by the presence of a condensed protein regime between two critical anion concentrations, thus creating a window of mutual miscibility.^16−18^ The physical principles that cause RC have been described by the re-entrant liquid condensation (RLC) model,^14,19,20^ and according to this model, protein condensation is caused by the binding of oppositely charged ions to the protein surface, resulting in neutralization of the long-ranged electrostatic interactions, allowing the formation of short-ranged ion-bridging attractive forces between the proteins.^14,19^ At higher concentrations of ions, the RLC model describes the decondensation process as being driven by screening and consequent inversion of the protein charge.^15,21,22^ Two critical parameters can be used to describe the RC process, the ion concentration at the condensation and at the decondensation boundaries, (C*) and (C**), respectively.^23^

RC has also been observed in other oppositely charged tertiary mixtures including multivalent anions in the presence of cationic proteins, cationic proteins in RNA solutions, and DNA in solutions containing multivalent cations.^24−27^ Recently, Bye and Curtis have shown that the polyvalent anions, pyrophosphate (PP) and tripolyphosphate (TPP), where SPP and STPP refer to the sodium salts, respectively, were able to induce the RC of the cationic protein lysozyme.^28^ In agreement with the RLC model, with increasing concentrations of PP and TPP, charge neutralization followed by charge inversion of the protein was observed.^28^ Interestingly, the polyvalent anion citrate, which has similar charge and size as PP, did not induce the RC of lysozyme, despite its ability to neutralize and subsequently invert its charge,^28^ which suggests that the RLC model is limited in ability to fully explain the condensation process observed in biological systems. This limitation stems from the fact that the RLC model considers only electrostatic interactions between the proteins and the ions. However, in addition to accumulation via electrostatic interactions, counterions can also be coordinated in a specific manner by charged residues.^27,29−32^ Hence, different anions may have different affinities with proteins, despite their similar charges. Subsequently, other, more specific, attractive interactions between the polyphosphate ions and lysozyme must be considered to understand the RC process observed.

The polyvalent anions, PP and TPP, are di- and tripolyphosphates, whereas citrate contains carboxyl groups, indicating the possibility that interactions between lysozyme and phosphate may play a key role in the RC process. Several proteins bind to DNA through interactions between the phosphate backbone of the DNA and positively charged arginine (Arg) and lysine (Lys) residues.^33,34^ Despite having similar charges, the interaction between Arg–phosphate is considerably stronger than that of Lys–phosphate.^35^ This is because the side chain, guanidinium present on Arg, can form additional interactions with phosphate groups.^33,34^ The lysozyme’s cationic nature is in part due to an abundance of the solvent-exposed Arg. We hypothesize that the interactions between Arg and the phosphate groups present in TPP and PP may provide the basis for the additional specific attractive interaction that causes the RC of lysozyme by TPP/PP but not by citrate.

**Figure 1:**
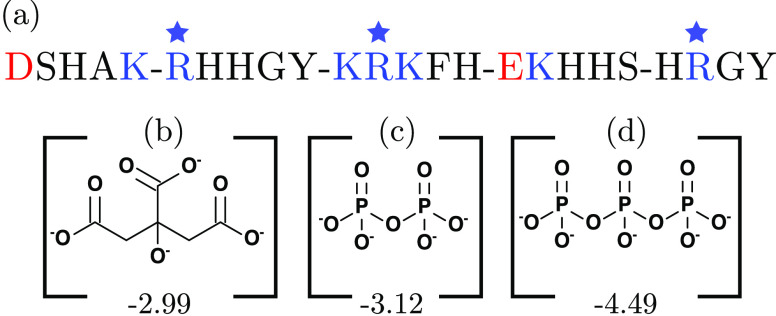
(a) Annotated histatin 5 sequence, where the negatively charged residues are colored red, the positively charged residues are colored blue, and arginine residues are indicated by a blue star. The molecular structure of the totally deprotonated states of the (b) citrate, (c) pyrophosphate, and (d) tripolyphosphate anions, respectively. The numbers below the structures correspond to the calculated net charge at infinite dilution of the anions at pH 8.4 used in this study.

In this paper, we investigate the role of Arg on the RC of the intrinsically disordered protein (IDP) histatin 5 (Hst5). Unlike globular proteins, IDPs do not adopt well-defined three-dimensional structures in solution but instead sample an ensemble of structures.^36^ Due to their innate flexibility and the high prevalence of charged residues, IDPs are often treated as polyelectrolytes. Previous studies have shown that IDPs also undergo RC in the presence of multivalent ions.^25,37,38^ Due to the polyelectrolytic nature of IDPs, the RC of this type of protein is more frequently referred to as coacervation.^39^ We selected Hst5 as a model protein for this study for several reasons:

- Hst5 is a cationic protein partly due to three Arg residues in the primary sequence.
- Arg residues are distributed evenly throughout the sequence, *i.e*., close to either termini and midsequence (see Figure 1a).
- The unfolded nature of Hst5 results in a high solvent accessibility of all residues within the sequence.
- Hst5 is well characterized by both experimental and computational techniques.^40−42^
- RC of IDP solutions are less studied, and a comparison with globular proteins may prove insightful.

The combination of distribution, frequency, and solvent accessibility of Arg within Hst5 yields an ideal model system to study the impact of Arg on the RC process in the presence of the polyvalent anion citrates, PP and TPP (see Figure 1b–d). By determining the phase behavior of Hst5 variants, which maintain the net positive charge but differ in the amount and distribution of Arg, we observe that while long-ranged electrostatic interactions drive the process of RC, specific Arg–phosphate interactions determine the location of both the condensation and decondensation boundaries. Comparison with lysozyme reveals similarities between the RC process observed, despite the differences between size, charge, and disordered versus globular structure of the proteins. We hypothesize that these differences can be accounted for based on the increased net charge and the higher content of Arg in lysozyme. By utilizing small-angle X-ray scattering (SAXS) and computer simulations, we observe that specific Arg–phosphate interactions result in a compaction of Hst5 at anion concentrations above the decondensation boundary. Altogether, our results indicate not only the importance of specific Arg–phosphate interactions in regulating RC but also provides insight for the rational modification of protein sequences to either promote or inhibit RC.

## Experimental Section

### Hst5 Variants

**Figure 2:**
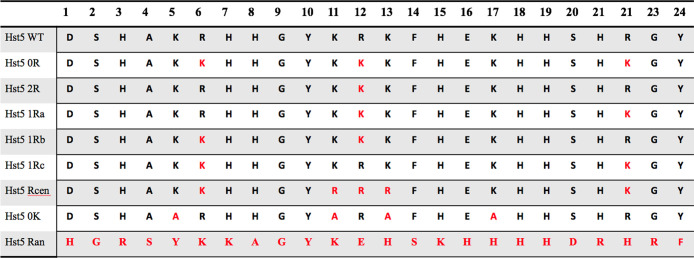
Variants of histatin 5 used in this study. Histatin 5 WT represents the wild-type sequence; modifications to the sequence are indicated by red font.

A selection of Hst5 variants was designed and synthesized by TAG (Copenhagen) with a purity between 97.0 and 99.0%. The variants used are listed in Figure 2.

### Sample Preparation

To remove any residual salt and contaminants, the protein was dialyzed exhaustively against Milli-Q water, using a dialysis membrane with an MWCO of 500 Da (BioTech). The resulting solution was then freeze-dried and stored at −20 °C. Prior to experimental measurements, the freeze-dried powder was dissolved in, nd further dialyzed against the buffer. The concentration of the protein solutions was measured by UV absorption at 280 nm using the extinction coefficient previously determined for Hst5 by amino acid analysis and mass spectroscopy (2560 M^–1^ cm^–1^). The ionic strengths of the prepared solutions were calculated based on the molar concentration of all ions present in the solution according to
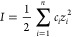
1where *I* is the total ionic strength and *c*_*i*_ and *z*_*i*_ are the concentration and charge of each ion constituent of the solution, respectively.

### SAXS Experiments

SAXS experiments were performed at the B21 beamline at the Diamond synchrotron (Oxford U.K.) and at the BM29 beamline at the ESRF synchrotron (Grenoble, France).^43,44^ At both B21 and BM29, sample delivery and measurements were performed using the automated BioSAXS robot.^45^ Prior and after each sample measurement, the corresponding buffer from dialysis was measured and averaged. Sequential sample frames were investigated for radiation damage prior to averaging and consequent subtraction of the buffer. Standards of known concentration and molecular mass were measured to calibrate the forward scattering angle (*I*_0_).

### SAXS Analysis

The buffer-subtracted averaged frames were analyzed for the presence of aggregation through investigation of the low-*q* region. The Guinier approximation was applied to extract the radius of gyration (*R*_g_) and t *I*_0_, from the scattering data using the limits *q*·*R*_g_ < 0.8. Measurements of a known standard protein (bovine serum albumin (BSA)) were used to calibrate *I*_0_ to obtain the association number *(N*_ass_.)

### Protein Precipitation Measurements

Protein precipitation measurements were completed using the method of Bye et al.^28^ In brief, the lyophilized Hst5 protein was dissolved in water and dialyzed exhaustively against Milli-Q H_2_O, followed by dialysis against the buffer three times for 12 h, which consisted of 10 mM Tris at pH 8.4. The concentration of the resulting solution was determined through UV measurements at 280 nm. Solutions were then made by addition of the stock protein to fresh buffer to which stock solutions of the polyvalent anions were added, yielding the desired anion concentration. The samples were incubated at room temperature on a shaking platform for 2 h. After incubation, the samples were centrifuged (10 000 rpm for 5 min), followed by a further incubation of 1 h, and thereafter centrifuged (10 000 rpm for 5 min) again. Thereafter, the concentration of the supernatant was directly measured using UV absorption. Turbidity of the same samples was monitored by UV measurements of the absorbance at 600 nm.

### ζ-Potential Measurements

ζ-Potential measurements were performed on a Malvern Zetasizer Nano ZS (Malvern Instruments Ltd., Malvern, U.K.) using DTS1070 folded capillary cells (Malvern Instruments Ltd., Malvern, U.K.). Prior to measurements, the samples were passed through a 0.22 μm filter (Whatman Syringe filters). The temperature was kept constant at 25 °C, and the samples were equilibrated at the set temperature for 180 s. For these experiments, an F(ka) of 1.5 (Smoluchowski’s approximation) and the dielectric constant of water, at the selected temperature (25 °C), were used. Three independent repeats were performed, and the results were averaged.

### Circular Dichroism

CD spectra in the far-UV region (190–260 nm, depending on the absorbance of the sample) were obtained using a Jasco J-7715 CD spectrometer, where the temperature was controlled using a PTC-348WI Peltier control system (Hachioji, Tokyo, Japan). The samples of Hst5 were prepared by dialyzing the protein powder against Milli-Q H_2_O (three changes) followed by dialysis against the specific buffer (three changes). The dialysis was completed in dialysis tubing with a 500 Da molecular weight cutoff (Biotech), performed at room temperature while stirring continuously. The samples were subsequently diluted in the dialysis buffer yielding a final protein concentration in the range of 0.2–0.25 mg/mL. Prior to each measurement, the samples were filtered through a Millex-GV filter with a 0.22 μm pore size (Merck Millipore Ltd., Ireland) into a quartz cuvette with a 0.1 cm path length and a total volume of approximately 400 μL. The temperature was set using the Peltier system and allowed to equilibrate for 10 min. For all measurements, a scan rate of 20 nm/min was used, with a 2 s response time and a 2.0 nm bandwidth. Each temperature measurement consisted of an average of five scans. The background spectra, consisting of the dialysis buffer, were collected under similar conditions for each temperature and subtracted from the sample spectra.

### Coarse-Grained Model

A coarse-grained model, previously applied to a range of IDPs, was used in this study.^6,46,47^ Despite the granular nature, this model provides excellent agreement with more detailed atomistic approaches and has been widely used.^6,40,46,48^ In the implemented model, individual amino acids are described by hard spheres connected by harmonic bonds, where the spheres mimic the excluded volume of the amino acid, including the hydration layer. Both the N- and C-termini were treated explicitly and included to account for their charge, while each sphere, or “bead”, had the charge of the corresponding amino acid. The bead radius was set to 2 Å, providing a realistic contact separation between a short-ranged attractive interaction and electrostatic interactions. The total potential energy of the system contained both bonded and nonbonded contributions and is described by
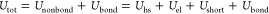
2where the nonbonded energy was assumed to be pairwise additive according to
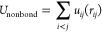
3where *r*_*ij*_ = |*R*_*i*_ – *R*_*j*_| is the center-to-center distance between two monomers/amino acids, and *R* refers to the coordinate vector. The excluded volume was taken into account through the hard-sphere potential *U*_hs_
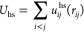
4which is summed up over all amino acids. The hard-sphere potential between beads in the model is given by
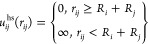
5where *R*_*i*_ and *R*_*j*_ denote the bead radii. The electrostatic interactions were modeled by an extended Debye–Hückel potential according to
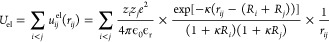
6where *e* is the elementary charge, *κ* denotes the inverse Debye screening length, ϵ_0_ is the vacuum permittivity, and ϵ_r_ is the relative dielectric constant of water. The short-ranged attractive interaction between the monomers was included through an approximate arithmetic average over all amino acids
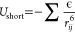
7where ϵ reflects the excess polarizability of the amino acid and thus sets the strength of the interaction. The bonded interaction is described by a harmonic potential
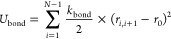
8where *r*_*i,i*+1_ denotes the distance in Ångstrom between two connected beads with the equilibrium separation *r*_0_ = 4.1 Å, where *N* denotes the number of amino acids in the protein. The force constant was set to *k*_bond_ = 0.4 N/m. Interactions between Arg residues and polyphosphate ions were modeled explicitly by representing the polyvalent ions as hard spheres with equivalent radius to the amino acids. A short-ranged attractive interaction was added between Arg and the polyvalent ion, using eq 6. The interaction strength, ϵ, was set to 8.0 × 10^4^ kJ Å^6^/mol, giving an attractive potential of 8.0 *kT* at the closest contact.

### Monte Carlo Simulations

The equilibrium properties of the systems were obtained by applying Metropolis Monte Carlo simulations^49^ in the canonical ensemble, i.e., the number of particles, the volume, and the temperature were constant. The protein chains were placed in a box with lengths of 250 Å with periodic boundary conditions in the *x-*, *y-*, and *z*-directions. The effect of box volume was investigated and none was observed. Long-ranged Coulomb interactions were truncated using the minimum image convention. In total, four different types of displacements were allowed: translational displacements of a single amino acid, pivot rotations, translation of the entire protein, and a slithering move. The probability of the different moves was weighted so that single-particle moves occurred 20 times more often than the other three. The protein chains were initially placed in the box in a random configuration, and an equilibration run of 5 × 10^5^ steps was performed. The production run consisted of a further 1 × 10^6^ steps. The simulations were performed using the integrated Monte Carlo/molecular dynamics/Brownian dynamics simulation package Molsim.^50^

## Results and Discussion

### Reentrant Condensation of Hst5 by the Polyvalent Anion TPP

**Figure 3:**
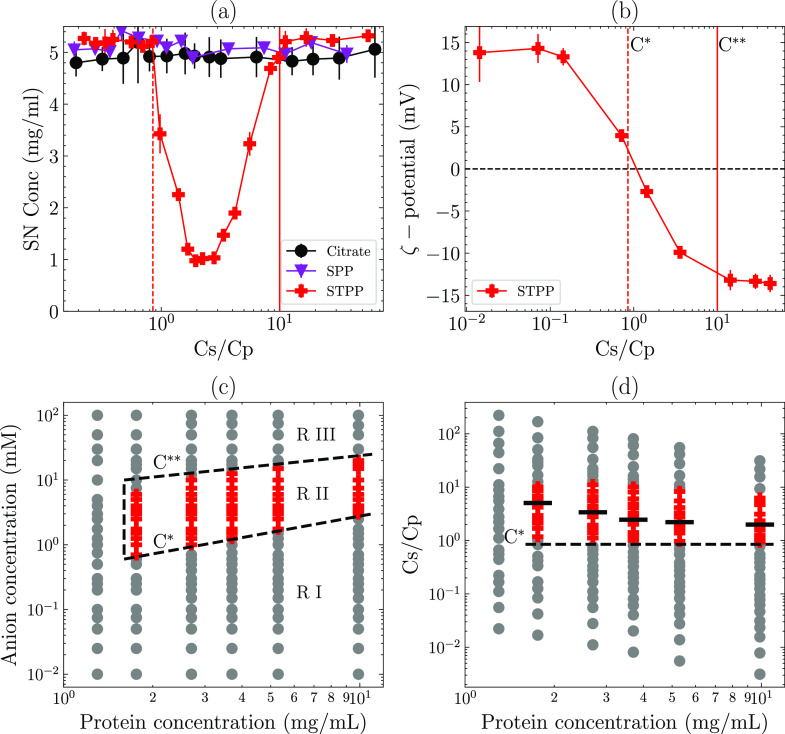
Histatin 5 undergoes re-entrant condensation and charge inversion in the presence of TPP. (a) Precipitation experiments performed at a fixed protein concentration of 5.2 mg/mL with varying concentrations of the anion indicated, performed at pH 8.4, depicting the concentration of the supernatant (SN) as a function of the anion-to-protein ratio. The dashed vertical line and solid line represent the location of C* and C**, respectively, in the Hst5/TPP solution. (b) Electrophoretic mobility of histatin 5 in the presence of varying concentrations of TPP, depicted as a function of the anion-to-protein ratio. The horizontal dashed line represents ζ-potential = 0, and the vertical dashed lines represent the C* and C** shown in Figure 1a. (c) Phase diagram of histatin 5 at the indicated protein concentrations in varying concentrations of TPP, where the C* and C** are indicated by horizontal dashed lines. The vertical dashed line represents the merge of C* and C** at low protein concentrations. RI, RII, and RIII correspond to regions below, between, and above C* and C**, respectively. Gray circles indicate no change in the concentration, whereas red crosses indicate a decrease in the soluble protein concentration (less than 95%). (d) The same data are shown in (c) as a function of the anion-to-protein ratio. Here, the solid horizontal lines represent the point of maximum precipitation.

The ability of TPP to induce the RC of Hst5 was assessed by preparing solutions containing a fixed concentration of Hst5 (5 mg/mL) with varying concentrations, ranging from 0.02 to 100 mM, of the selected polyvalent anions. The instantaneous formation of a white precipitate was observed in the solutions that contained between 1.1 and 11 mM TPPs. The visual observations were confirmed by measurements of the soluble protein retained in the supernatant after centrifugation, which is shown in Figure 3a, against the ratio of anions per protein molecule (*C*_s_/*C*_p_) in the initial solution. No effect was observed in solutions containing either PP or citrate. However, in response to increasing TPP concentration, Hst5 displays characteristic RC phase behavior, undergoing sequential liquid-to-solid and solid-to-liquid phase transitions. The turbidity of the solutions did not change for the solutions containing PP or citrate. The turbidity of the SPP solutions showed the same concentration dependence as observed for the precipitation measurements. ζ-Potential measurements revealed that the charge of the protein steadily decreases and eventually changes sign with increasing TPP concentration, as shown in Figure 3b.

The phase diagram of Hst5 in the presence of TPP was determined by performing precipitation experiments at different protein concentrations (see Figure 3c). Both the condensation and decondensation boundaries, C* and C**, respectively, are linearly dependent on the protein concentration. Strikingly, when the phase diagram is depicted as a function of the number of anions per protein (*C*_s_/*C*_p_) as in Figure 3d, C* is located at an almost constant ratio of anion per protein, fluctuating from 1.1 to 0.9 from the lowest to the highest concentration investigated. Both C** and the point of maximum precipitation vary slightly with the protein concentration, where the latter ranges from *C*_s_/*C*_p_ = 2.5, at the lowest protein concentration, to *C*_s_/*C*_p_ = 2 at the highest. Irrespective of the starting protein concentration, at maximum precipitation, the protein concentration of the supernatant (SN) decreases to 1 mg/mL. Readers should notice that fresh solutions containing 1 mg/mL protein did not display protein precipitation under any of the TPP concentrations measured (see Figure 3c).

### The locations of both C* and C** Are Determined by the Presence of Arg within the Sequence

**Figure 4:**
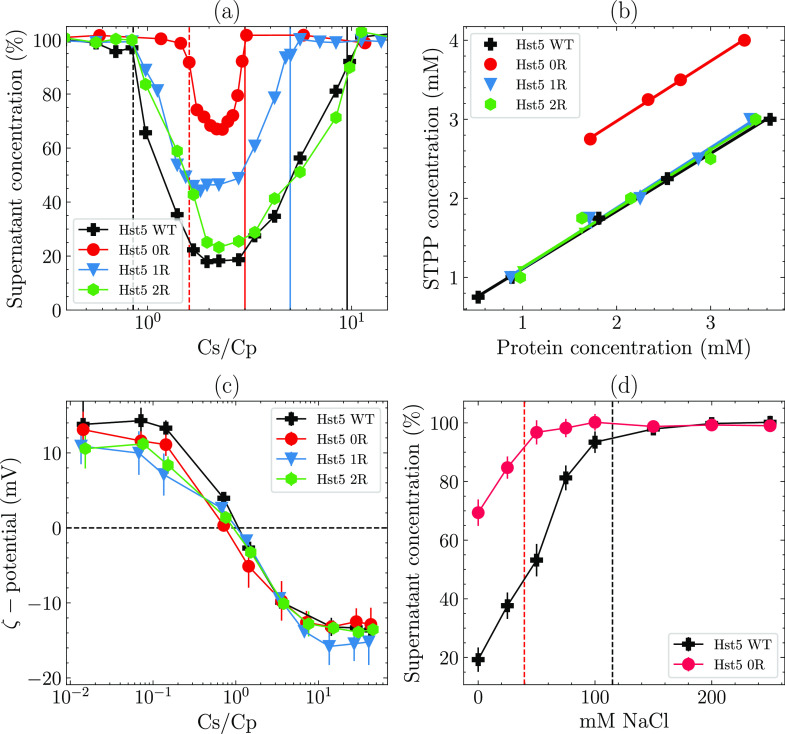
Comparison of the RC behavior of histatin 5 with variants of constant linear charge density and variant number of Arg. (a) Precipitation experiments performed at a fixed protein concentration of 5.2 mg/mL shown for the indicated variants of histatin 5 as a function of the protein concentration remaining in the supernatant against the anion-to-protein ratio (*C*_s_/*C*_p_). Histatin 5 WT is shown for reference (same as Figure 2). Dashed and solid vertical lines depict the location of the C* and C** boundaries, respectively. (b) The location of the C* boundary, determined by precipitation experiments performed at the indicated protein concentrations; the linear fit shown is according to eq 9. (c) ζ-Potential measurements performed at the indicated anion concentration and fixed protein concentration shown for histatin 5 and variants. (d) Variation of the measured supernatant concentration upon the addition of NaCl measured at the TPP concentration corresponding to the maximum point of precipitation. The dashed line corresponds to the NaCl required to reach ionic strengths equivalent to those calculated in solutions at the C** boundaries depicted in (a).

To investigate the potential influence of short-ranged interactions between the Arg in Hst5 and the phosphate on TPP, variants of Hst5 with constant linear charge density, but with varying number of Arg were designed, by the substitution of Arg with Lys, in the wild-type (WT) sequence. Three different variants were initially designed, Hst5 0R, Hst5 1R, and Hst5 2R, containing zero, one, or two Arg residues, respectively. Precipitation measurements showed that all variants depicted RC behavior in the presence of TPP as shown in Figure 4a, where the precipitation measurements performed for Hst5 WT and Hst5 2R yielded similar results, indicating that the presence of two Arg residues is sufficient to mimic the RCP of the WT. Quantitatively, the concentration range for the phase-separated regime becomes narrower upon decreasing the Arg contents of Hst5 below two. Similarly, the amount of protein precipitated from solution also decreases with decreasing Arg content. Figure 4a displays C* determined for Hst5 and the variants at different protein concentrations. The C* boundary is located at the same *C*_s_/*C*_p_ concentration for all variants that contain at least a single Arg residue, while the complete depletion of Arg from the sequence increases the location of C* to a higher TPP concentration.

In solutions containing trivalent and polyvalent salts, the location of C* has previously been described using the empirical relationship^17^
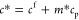
9eq 8 is valid when c* occurs at a critical value of bound ions per protein given by m* at a specific protein concentration (*c*_p_). The empirical relationship considers the free solution salt concentration (*c*^f^) in equilibrium with the protein–ion complex, when m* ions are bound. Table 1 lists the values of *c*^f^ and m*, obtained by determining C* at a range of protein concentrations (shown in Figure 4b). The molar amount of ions bound per protein at C* does not vary significantly between the variants, on average 0.7 ions per protein. We next calculated the charge contribution of ions bound to the protein, defined as the charge contribution in Table 1, by multiplying m* by −4 (the net charge of TPP); for each variant, the calculated contribution lies between 3.7 and 3.8. The free ion concentration does, however, differ between variants containing Arg and Hst5 0R, ranging from 0.35 to 1.456, respectively. Thus, the major difference between variants containing Arg and Hst5 0R is the ability of TPP to bind at lower concentrations when Hst5 contains at least a single Arg residue.

**Table 1:** Parameters Extracted from Fitting the Data from the Precipitation Diagrams, Shown in Figure 4b, by Using eq 9

	m*	**charge contribution**	***c***^**f**^
Hst5 WT	0.762 ± 0.04	3.81	0.355 ± 0.012
Hst5 2R	0.741 ± 0.03	3.80	0.379 ± 0.09
Hst5 1R	0.723 ± 0.04	3.61	0.384 ± 0.09
Hst5 0R	0.760 ± 0.09	3.70	1.456 ± 0.12

ζ-Potential measurements performed on the Hst5 variants (see Figure 4c) revealed similar charge inversion transitions as for Hst5 WT, confirming that it is not sufficient to fully describe the observed RC behavior. As previously highlighted, Arg is capable of mediating a stronger short-range multimodal interaction network with phosphate groups, compared to the similarly charged Lys. We determined experimentally whether this was a contributing factor in the RC of Hst5 by TPP by performing precipitation measurements of Hst5 and Hst5 0R with increasing ionic strengths, which should decrease the propensity to phase-separate due to electrostatic screening.^51^ The measurements were performed at TPP concentrations corresponding to the maximum point of precipitation (see Figure 4a). For both proteins, a decrease in precipitation with increasing ionic strength is observed, consistent with a process that is electrostatically driven as shown in Figure 4d. Increasing the Arg contents of Hst5 requires higher ionic strengths to completely inhibit protein precipitation. The ionic strengths of the solutions at the C** boundaries were evaluated, and the equivalent concentration of NaCl required to reach the same ionic strength was determined (see Figure 4d). Interestingly, the determined ionic strength is close to where the precipitation reaches zero, and the C** boundary is therefore located at ionic strengths where the interaction is screened. These results support the hypothesis that the interactions between Arg–TPP interactions are stronger than those of Lys–TPP, where the latter can be tuned by the ionic strength. Hence, electrostatic screening is responsible for the increased C** boundary observed with increasing Arg contents. Since C** is not affected by an increase from two to three Arg residues, we conclude that for Hst5 a maximum of two Arg residues participate in the RC process.

### RC of Hst5 by PP Depends on the Protein Charge and the Number of Arg Residues but Not on Their Sequence Distribution

**Figure 5:**
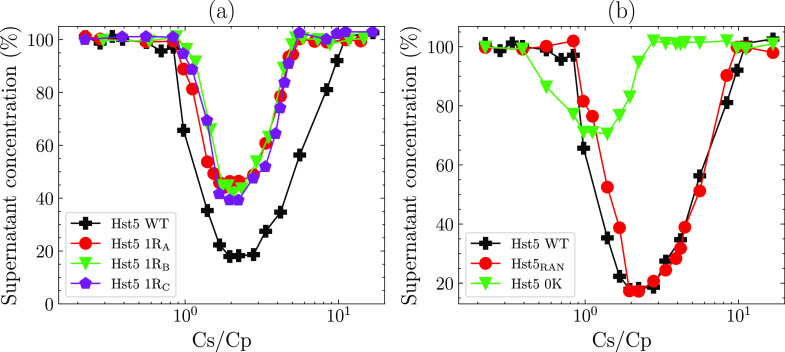
Precipitation experiments performed at a fixed protein concentration (5.2 mg/mL) with varying TPP concentrations, as a function of the supernatant concentration against the anion-to-protein ratio (*C*_s_/*C*_p_). (a) Histatin 5 variants containing only single Arg at different positions, keeping the linear charge density constant, compared with histatin 5 WT. (b) Randomized histatin 5 sequence and histatin 5 0K, where the charge of the sequence has been reduced by replacing lysine with alanine, compared with histatin 5 WT.

Both the position and the properties of the amino acids surrounding Arg residues may potentially influence the interaction with TPP. For example, for globular proteins, charged amino acids are often collectively described by charge-patch regions. To determine the significance of the position and local environment, precipitation experiments were performed on variants of Hst5 that maintained a single Arg residue close to the N-terminus (Ra), the middle of the sequence (Rb), or C-terminus (Rc), as shown in Figure 5a. No significant effect on the RC behavior was observed; hence, the position of the Arg and the properties of the surrounding amino acids have no significant influence on the location of C* or C**. This was further confirmed by determining the RC behavior of a randomized Hst5 sequence, which shows similar behavior to the WT sequence (see Figure 5b). In all of the experiments listed so far, the overall linear charge density of Hst5 has been conserved while varying the number of Arg. To determine the extent to which the protein net charge influences the RC process, a variant of Hst5 was designed that maintained the three Arg residues present in WT while substituting all four Lys for alanine (Hst5 0K), thereby decreasing the overall positive net charge .

Precipitation experiments of Hst5 0K show a decrease in C*, C**, and *C*_max_ and the total amount of protein precipitated from solution compared to that of Hst5 WT, as shown in Figure 5b. The shift of the whole RC regime to lower *C*_s_/*C*_p_ can be explained by the decreased net positive charge of Hst5 0K, which results in a reduction in the number of ions required to both screen the charge of Hst5 0K and invert the charge of the protein, compared to the WT and variants, where the protein charge is maintained. Together, these results confirm that the observed RC process depends on a combination of the protein net charge and the number of Arg present in the sequence.

### Solution Behavior of Hst5 and Variants above C**

**Figure 6:**
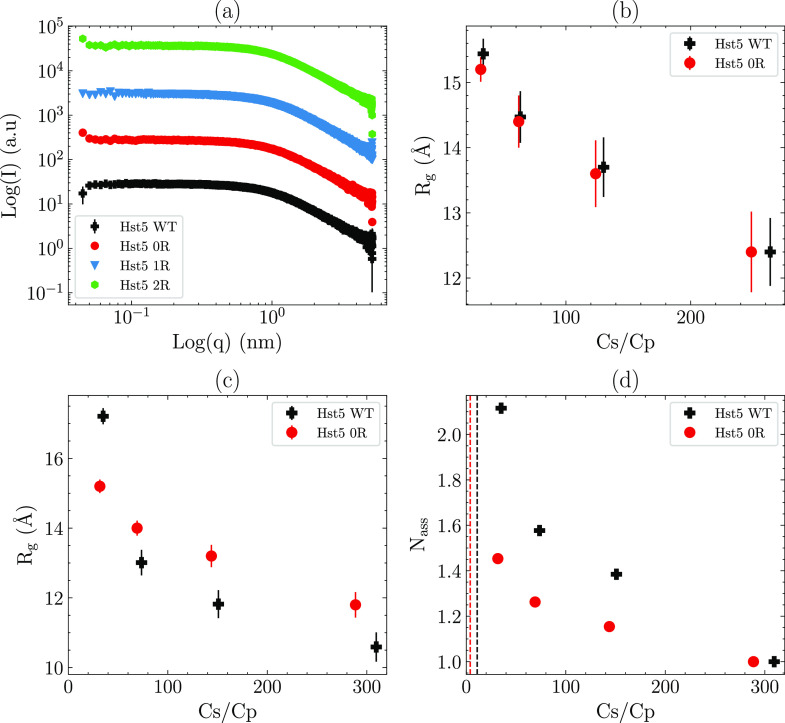
SAXS analysis of histatin 5 and variants performed at a fixed protein concentration (5 mg/mL). (a) SAXS spectra of histatin 5 and variants as indicated in 100 mM NaCl. (b) Radius of gyration (*R*_g_) determined from the SAXS spectra of histatin 5 and histatin 5 0R in citrate at varying *C*_s_/*C*_p_ ratios. (c) Radius of gyration (*R*_g_) determined from the SAXS spectra of histatin 5 and histatin 5 0R in TPP at varying *C*_s_/*C*_p_ ratios. (d) Association number (*N*_ass_) determined from *I*_0_ for histatin 5 and histatin 5 0R in TPP at varying *C*_s_/*C*_p_ ratios. The dashed lines represent the location of C** determined from the precipitation experiments.

To rule out any changes to the protein structure caused by varying the sequence of Hst5, SAXS spectra were collected in 100 mM NaCl. No changes in structural properties between Hst5 WT and the variants were visible (see Figure 6a).

**Figure 7:**
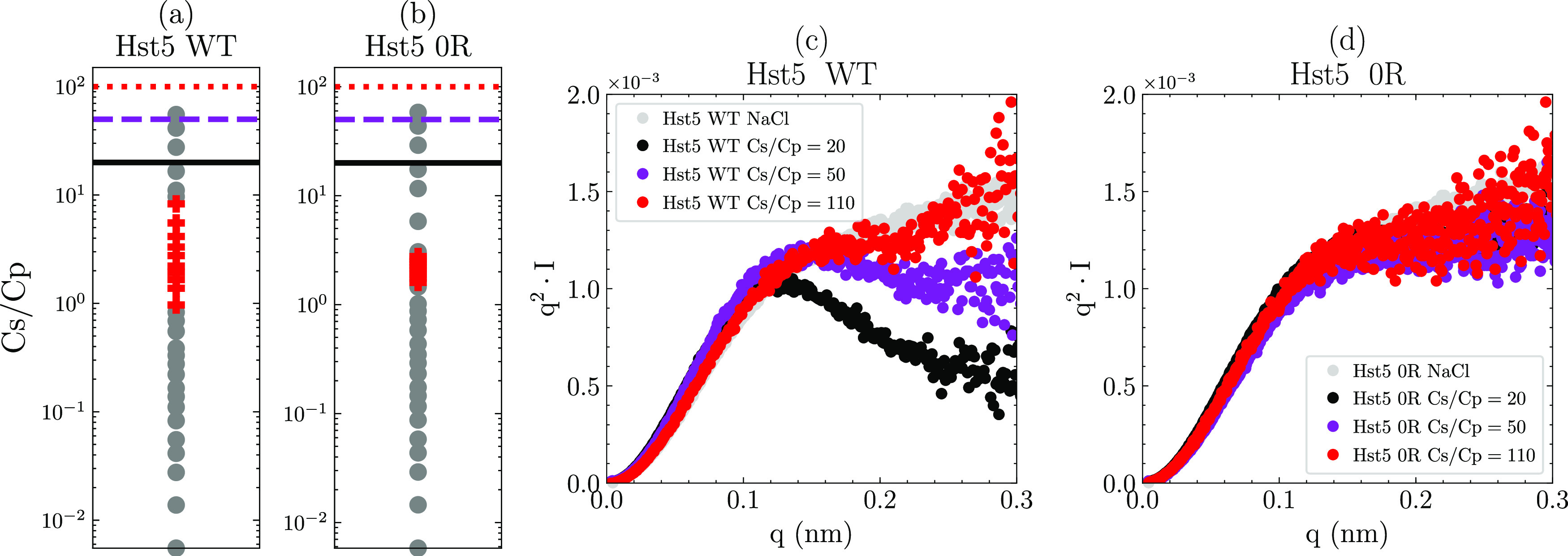
Comparison between the solution behavior of histatin 5 WT and histatin 5 0R in solutions containing TPP at various *anion per protein ratios (*C*_s_/*C*_p_).C*_s_/*C*_p_ ratios compared to NaCl SAXS data. (a) Precipitation results of histatin 5 WT at 5 mg/mL; data are the same as in Figure 2. (b) Histatin 5 0R precipitation results for histatin 5 0R. (c) Kratky plots of the histatin 5 SAXS data collected in TPP solutions at various *C*_s_/*C*_p_ ratios. (d) Kratky plots of the histatin 5 0R SAXS data collected at various *C*_s_/*C*_p_ ratios. The horizontal lines in (a) and (b) correspond to the *C*_s_/*C*_p_ ratios shown in (c) and (d).

Having established that specific Arg–phosphate interactions affect interprotein interactions of Hst5, we next investigated the influence of polyvalent anions on the intramolecular interactions of Hst5 above C** using SAXS. Figure 6b shows the *R*_g_ of Hst5 and Hst5 0R determined in the presence of citrate at varying *C*_s_/*C*_p_ ratios. Both of the peptides show the same tendency of reduction in *R*_g_ with increasing citrate concentration, caused by the formation of larger oligomers at low *C*_s_/*C*_p_, which indicates that even though no RC is observed in these solutions, clusters or oligomeric species are formed. Similar oligomeric behavior was also observed in the presence of TPP (see Figure 6c), although there is a discrepancy between Hst5 WT and Hst5 0R, caused by the tendency of Hst5 WT to form oligomers at lower *C*_s_/*C*_p_, as determined by r *N*_ass_, as shown in Figure 6d. The reader should note that the association number is higher in the TPP solutions, yet for several samples, the determined *R*_g_ is lower. The formation of larger clusters by Hst5 WT, compared to that of Hst5 0R, in TPP is not surprising considering that the C** boundary is located at higher *C*_s_/*C*_p_ for the WT sample (see the dashed lines in Figure 6d), indicating that even above C** the attractive interactions between Arg and TPP lead to the formation of higher-order oligomers compared to Hst5 0R. Similarly, although the association number is significantly higher for WT, the *R*_g_ is only higher at the lowest *C*_s_/*C*_p_ value measured. This can be explained by the fact that the stronger interactions between TPP and the Arg residues of Hst5 WT result in the compaction of the peptide chain. Comparison of the Kratky plots obtained at the highest *C*_s_/*C*_p_ ratio with those obtained at the lowest values (see Figure 7) shows that Hst5 WT forms larger oligomers that are more compact than those formed by Hst5 0R at low concentrations of TPP. Hence, the interprotein interactions persist above C**, although the clusters are soluble in nature.

### Influence of Polyvalent Anions on Conformational Properties of Hst5

**Figure 8:**
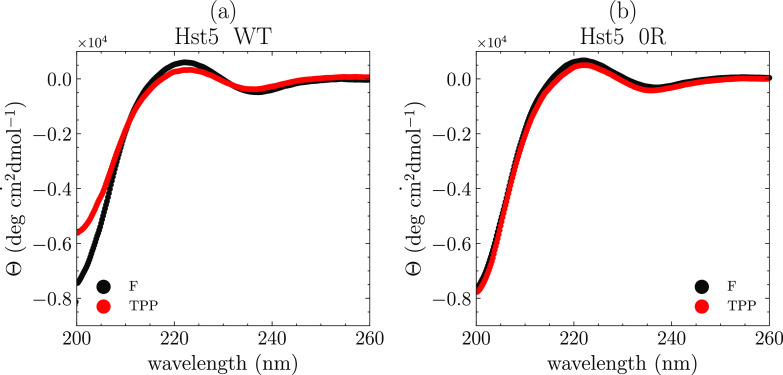
Circular dichroism spectra collected at 0.2 mg/mL in 100 mM of the indicated anion shown for (a) histatin 5 WT and (b) histatin 5 0R.

In contrast to globular proteins, IDPs lack a well-defined conformation in solution and instead sample an ensemble of conformations. Due to the polyelectrolytic nature of IDPs, the conformational ensemble is influenced by the presence of ions and other cosolutes.^52^ For example, divalent cations induce compaction of an anionic IDP, compared to monovalent salt, and theoretical studies have suggested that polyvalent ions may cause compaction of polyelectrolytes.^53,54^ According to Figure 6b,c, minor differences were observed between the *R*_g_ determined for Hst5 WT and Hst5 0R in TPP, but not citrate. To exclude that the formation of the secondary structure caused the compaction, circular dichroism measurements were performed at a low protein concentration (0.25 mg/mL) in the presence of TPP and compared with monovalent salt (see Figure 8). Only insignificant changes were observed between the two proteins; thus, the differences in *R*_g_ between the two anions were not caused by ion-induced folding.

**Figure 9:**
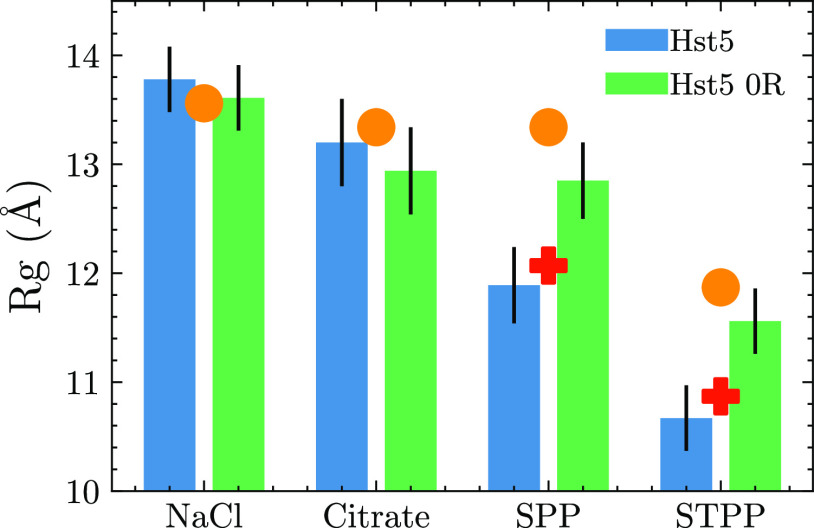
Radius of gyration (*R*_g_) determined from experimental SAXS for histatin 5 (blue bars) and histatin 5 0R (green bars), bead-necklace simulations (orange circles) and bead-necklace simulation with an added attractive interaction between arginine residues and the anions (red plus).

More detailed information about the influence of the anions on the conformational ensemble was obtained by SAXS measurements performed at protein concentrations of 1 mg/mL, below the C* boundary, in the presence of the polyvalent ion TPP. Both *R*_g_ and *I*_0_ were extracted by the Guinier analysis of the low-*q* scattering, where the *I*_0_ values did not show any evidence of interparticle effects at low protein concentrations. The *R*_g_ values determined are shown in Figure 9. A clear dependence of *R*_g_ on the added salt can be observed, where TPP yields the most compact conformations and NaCl the most extended, which is in agreement with what one might expect based on the ability of higher charged ions to induce stronger structural changes of polyelectrolytes.^53,54^ It has previously been shown that the structural properties of Hst5 can be accurately captured by computer simulations using a coarse-grained model, taking into consideration the charges of each amino acid and the properties of the buffer solution. The simulations were performed by treating the anions as spheres with charges corresponding to the charge of each polyvalent anion. The simulations yielded results that were in agreement with the experimental SAXS data for both Cl and citrate anions (Figure 8); however PP, which has a similar charge to citrate, gave a larger *R*_g_ than expected from the experimental SAXS data. A similar overestimation of *R*_g_ was also observed for TPP. The results suggest that the interaction between citrate and Hst5 agrees well with that expected of an interaction between the anion and protein based on electrostatic interactions, but those of PP and TPP did not. Based on the possible interaction between Arg and phosphate, an extra attractive term between Arg and the anion was included in the model to mimic specific nonelectrostatic interactions. These simulations yielded more accurate results compared to the experimental data (see Figure 8). SAXS data was also collected for Hst5 0R under similar conditions, where comparison between the SAXS and simulations showed a closer agreement with the purely electrostatic model, which indicates that the properties of the polyvalent anion influence not only the RC behavior but also the properties of the protein conformational ensemble when Arg residues reside within the sequence.

### Comparing the RC Behavior of Hst5 with the Globular Lysozyme Protein

**Figure 10:**
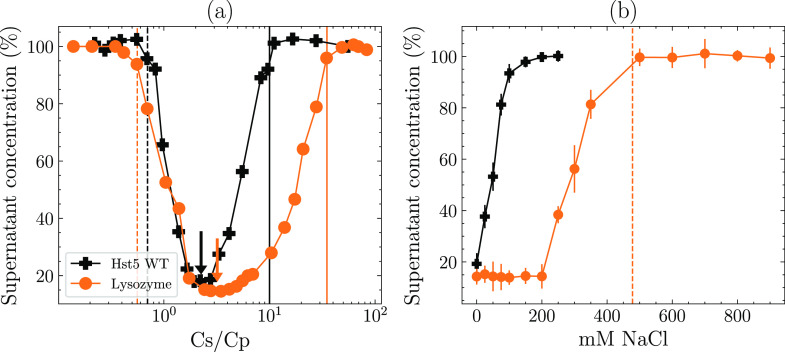
Comparison between the rentrant condensation behavior of histatin 5 and lysozyme. (a) Supernatant concentration as a function of the anion per protein ratio (*C*_s_/*C*_p_). The dashed line depicts the C* boundary, the arrows indicate the location of *C*_max_, and the solid vertical lines depict the C** boundary. (b) Concentration of the supernatant at the TPP concentration corresponding to *C*_max_, performed at varying NaCl concentrations. The orange dashed line represents the addition of NaCl required to reach the ionic strength of the solution corresponding to C**.

We have shown that the IDP Hst5 can, like globular proteins, undergo RC despite their different conformational properties. The RC behavior of the globular protein lysozyme, in the presence of TPP, has previously been characterized at pH 9.0.^28^ The precipitation experiments were repeated at the pH used for Hst5 in this study, pH 8.4 (see Figure 10a), and compared with Hst5 solutions containing the same molar concentration of protein. The observed maximum point of precipitation is 1.5 times higher for lysozyme compared to that of Hst5. For the lysozyme measurements, C* is located at *C*_s_/*C*_p_ = 0.5, lower than observed for Hst5, which is *C*_s_/*C*_p_ = 0.9, while C** is located at much higher *C*_s_/*C*_p_ ratios compared to Hst5. Indeed, inhibiting the precipitation at *C*_max_ of lysozyme solutions required fourfold as much NaCl, compared to Hst5 (see Figure 10b). Similarly to Hst5, we observed that the concentration of NaCl required to inhibit precipitation corresponded to the same ionic strength as observed in solutions at the C** boundary. Combined, there are some differences between lysozyme and Hst5 RC. We propose that these differences can be explained based on the increased charge, size, and number of Arg residues present on lysozyme combined with the globular nature of the protein. However, despite these differences, fundamentally similar Arg–phosphate interactions contribute to the RC of lysozyme in PP/TPP solutions.

The ionic strengths of the solutions at C**, at the same protein concentrations, are listed in Table 2. Based on the Hst5 results, we can speculate at the ionic strength contribution per Arg residue. Going from Hst5 1R to Hst5 2R, it requires almost double the ionic strength to reach C**. If we make assumptions based on these two data points, we speculate that four to five combined Arg residues of lysozyme may participate in the interparticle attractive interactions. This number does not seem unrealistic as each lysozyme contains 10 solvent-exposed residues. However, we emphasize that this is based on extrapolation from two data points. In this estimation, we ignore the contribution of the increased ionic strength found at C** of lysozyme solutions; thus, the number of Arg residues is probably higher than the estimated 4–5.

**Table 2:** Calculated Ionic Strengths of the Solutions at the C** Boundary^a^

	***I*****at**C**(mol/L)	**I/Arg**
Hst5 0R	0.04	
Hst5 1R	0.08	0.08
Hst5 2R	0.16	0.08
Hst5 WT	0.16	0.08
lysozyme	0.36	

a For histatin 5 with one or more Arg residues, the ionic strength divided by the number of Arg residues is listed (I/Arg).

The observed shifts of C*, *C*_max_, and C** to different *C*_s_/*C*_p_ values (shown in Figure 10a), in lysozyme/TPP solutions compared to those in the Hst5/TPP solutions, can be explained by the contribution of both electrostatic and specific interactions to the RC behavior of the respective proteins. The decrease in C* is caused by the increased attractive interactions between lysozyme and TPP due to the higher net positive charge of lysozyme compared to that of Hst5. The increased positive net charge of lysozyme may also explain why previous studies observed RC in lysozyme/PP solutions that we did not observe in Hst5/PP solutions.^28^ There is also a modest increase in *C*_max_ (see the arrows in Figure 10) caused by the higher ionic strength required to neutralize the more highly charged lysozyme protein compared to that of Hst5. Finally, the increase in C** observed in the lysozyme solutions, compared to that of Hst5, is due to a combination of the higher net charge of lysozyme and the increased abundance of Arg residues on the surface of the lysozyme protein.

## Conclusions

RC caused by the charge inversion of cationic and anionic proteins in the presence of oppositely charged multivalent ions is thought to be a universal phenomenon.^55^ However, recent experimental studies have shown that in the case of polyphosphate anions, the process cannot be described by electrostatics alone.^19,28^

**Figure 11:**
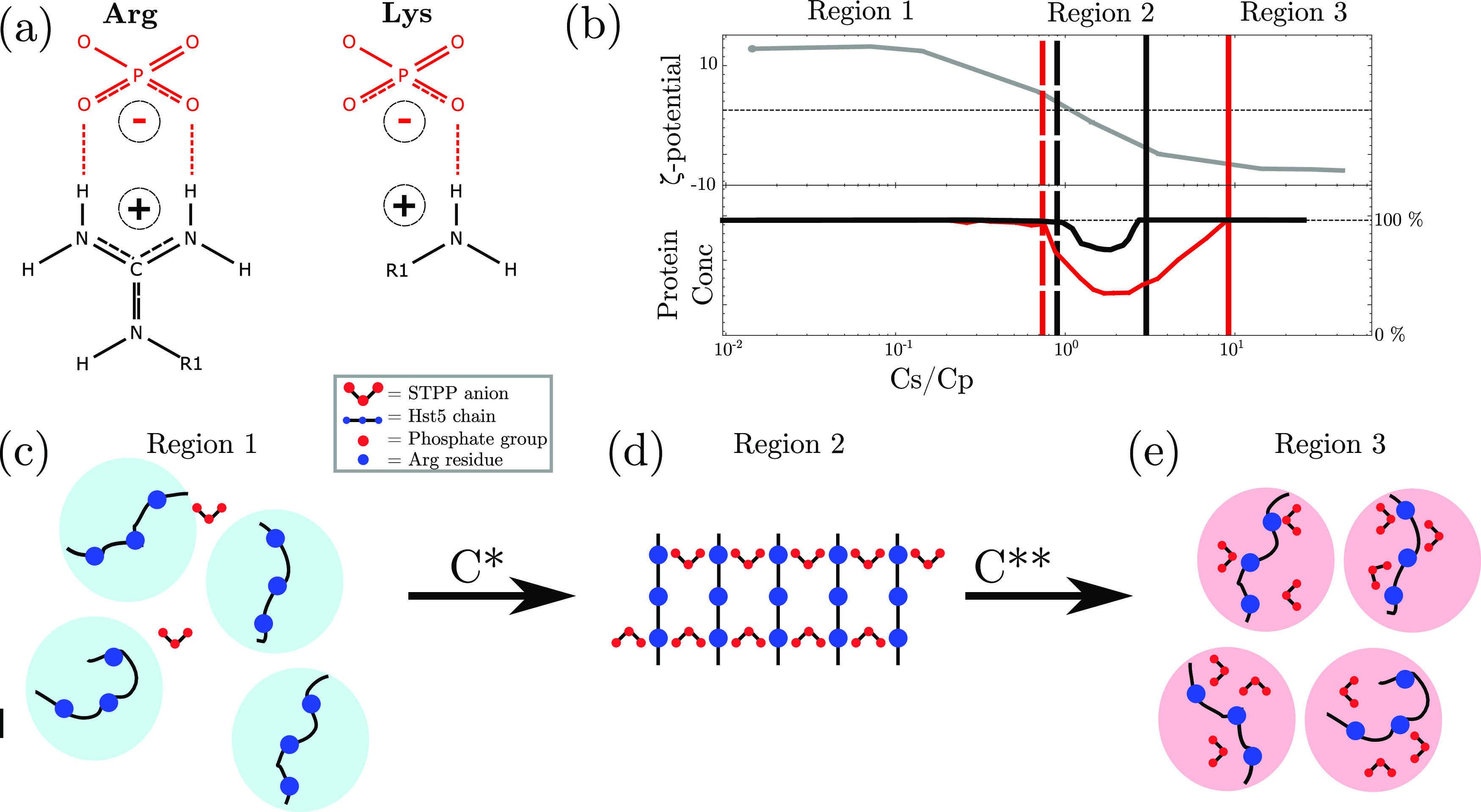
Schematic depiction of results. (a) Arginine can form additional attractive interactions with the phosphate groups, such as those present on tripolyphosphate (TPP, compared to Lys. (b) Top: ζ-potential measurements depict a similar charge inversion for both histatin 5 WT and histatin 5 0R in the presence of TPP. Bottom: comparison of the reentrant condensation (RC) behavior between histatin 5 WT (red) and histatin 5 0R (black). Distinct differences in both C* and C** are observed despite the similar net charges of the proteins. C* is located at lower *anion per protein ratio (*C*_s_/*C*_p_)* ratios in histatin 5 WT due to the additional attractive interactions between Arg and phosphate, while C** is located at higher ratios due to the additional ionic strength required to screen out the interactions. (c–e) Schematic depiction of the RC process of histatin 5. At low *C*_s_/*C*_p_, histatin 5 maintains its net positive charge inducing repulsion. Between C* and C**, ion-bridging is observed causing cross-linking effect and consequent precipitation, whereas above C**, the charge of histatin 5 is inverted, and long-ranged electrostatic repulsion is reintroduced.

We hypothesized that specific Arg–phosphate interactions (Figure 11a) may provide additional attractive interactions required to explain the RC of lysozyme in the presence of TPP/PP solutions. We have shown that Hst5 can, like lysozyme, undergo RC in the presence of TPP (Figure 10b). In a similar manner to lysozyme, the charge of Hst5 decreases, passes through zero, and becomes negative upon increasing TPP concentrations (see Figure 11b). Quantitatively, the concentration range for the RC regime is more narrow for Hst5 compared to that for lysozyme, caused by an increase in C* and a decrease in C**. Measurements of variants that differed in the amount of Arg indicated that the locations of both C* and C** are determined by the number of Arg residues, as shown in Figure 10b. By increasing the ionic strength of the solutions at the maximum point of precipitation by the addition of NaCl, we showed that the location of C** is determined by the ionic strength of the solution and thus can be described by an electrostatic-screening effect, while C* only increased upon complete depletion of Arg from the sequence. We propose the mechanism depicted in Figure 11c–e: at low *C*_s_/*C*_p_, the protein is positively charged and molecules repel each other, increasing the anion concentration, which results in ion binding to the protein surface. When Arg residues are present, this occurs at a lower *C*_s_/*C*_p_ due to the increased strength of interaction compared to Lys (Figure 11a); at *C*_s_/*C*_p_ ratios above C**, the charge of the protein is inverted, reintroducing repulsive interactions.^17,19,24^ The presence of C** is determined by the ionic strength required to screen the Arg– and Lys–phosphate interactions. This requires a higher concentration for the former and thus C** is located at a higher *C*_s_/*C*_p_ ratio.

The interactions between Arg and TPP were further characterized by SAXS. While Hst5–citrate interactions gave an *R*_g_ close to those determined by simulations that only considered the charge of the anions, the corresponding numbers for TPP and PP were overestimated. The addition of an extra attractive interaction between Arg and the anion provided more comparable results, indicating that specific Arg–phosphate interactions not only determine the RC behavior but also influence the conformational ensemble. The RC behavior observed in protein–polyphosphate solutions can therefore be explained by a combination of charge inversion and additional Arg–phosphate interactions.

The implication of RC-induced polyphosphates may have more general applications in the area of IDP aggregation. Reduction of solubility of both IDPs and globular proteins by polyphosphates has been shown to control fibril formation.^56^ This topic is of special interest in the field of amyloid proteins, where polyphosphates have been shown to influence the morphology of amyloid fibrils formed.^57,58^
